# Case Report: Use of the over-the-scope clipping system in a trauma patient for a large bowel anastomotic leak

**DOI:** 10.3389/fsurg.2026.1695835

**Published:** 2026-05-20

**Authors:** Thamer Nouh

**Affiliations:** Trauma & Acute Care Surgery Unit, Department of Surgery, College of Medicine, King Saud University, Riyadh, Saudi Arabia

**Keywords:** anastomotic leakage, case report, gastrointestinal surgery, OTSC, trauma

## Abstract

Anastomotic leakage is a relatively common and serious complication following gastrointestinal surgery. Traditionally, management has relied on surgical re-intervention with fecal diversion, often followed by a secondary reversal procedure. In recent years, the over-the-scope clip (OTSC) system, originally developed for gastrointestinal perforations and bleeding, has emerged as a minimally invasive alternative for selected cases of anastomotic leakage. I report the case of a 47-year-old trauma patient who developed a persistent colonic anastomotic leak following large bowel resection. After failure of conservative management, the defect was successfully closed using the OTSC system, with complete resolution and no post-procedural complications.

## Introduction

Anastomotic leakage is a serious and potentially life-threatening complication following gastrointestinal surgery, associated with significant morbidity, mortality, and prolonged hospitalization. Reported incidence rates range from 2.8% to 30%, depending on the anastomotic site, surgical technique, and patient-related risk factors (1, 2).

Historically, management has relied on surgical re-intervention aimed at sepsis control, frequently involving fecal diversion with a diverting ileostomy, followed by a subsequent procedure to restore intestinal continuity (2, 3). However, advances in interventional endoscopy have introduced less invasive therapeutic alternatives.

The over-the-scope clip (OTSC) system was initially developed for the management of gastrointestinal bleeding, perforations, and fistulas. More recently, it has gained attention as a treatment option for anastomotic leaks due to its ability to achieve full-thickness tissue approximation. Its favorable efficacy and safety profile make it a promising option in selected patients, particularly those considered poor candidates for surgical re-intervention (4–6).

## Case presentation

A 47-year-old male presented to the Emergency Department after being run over by a motor vehicle in a roadside accident. Initial assessment revealed hemodynamic instability, manifested by hypotension (blood pressure 85/50 mmHg) and tachycardia (heart rate 125 bpm), necessitating immediate crystalloid resuscitation. A focused assessment with sonography for trauma (FAST) demonstrated free intraperitoneal fluid in Morison's pouch and the pelvis, suggesting intra-abdominal injury.

Following initial stabilization, the patient underwent emergent exploratory laparotomy. Intraoperative findings included a minor splenic injury; a 70-cm segment of devascularized small bowel, which was resected with primary anastomosis; a small sigmoid colon perforation managed by segmental resection with side-to-side stapled colo-sigmoid anastomosis; rupture of the urinary bladder dome repaired primarily; and a degloving injury of the anterior abdominal wall treated with debridement and wound care.

A 24-French closed-suction drain was placed in the pelvis adjacent to the colo-sigmoid anastomosis for prophylactic monitoring of potential leakage. Additionally, the patient sustained bilateral tibial fractures stabilized with intramedullary nailing.

Postoperatively, the patient was admitted to the intensive care unit and transferred to the surgical ward on postoperative day 5. Shortly thereafter, increased high-volume enteric output was observed from the intra-abdominal drain.

A contrast-enhanced CT scan demonstrated extravasation of orally administered contrast material at the colo-sigmoid anastomosis, associated with a contained heterogeneous presacral fluid collection measuring 4.5 × 3.0 cm, consistent with a localized abscess. Ultrasound-guided percutaneous drainage was performed.

Despite conservative management—including bowel rest, microbiology-guided broad-spectrum intravenous antibiotics, total parenteral nutrition, and continued drainage—the anastomotic leak persisted. Repeat CT imaging on postoperative days 18 and 28 confirmed ongoing contrast extravasation without radiological reduction of the associated collection.

Based on persistent high-volume (>200 mL/day) drain output and radiological evidence of ongoing leakage, conservative management was deemed unsuccessful.

**Figure 1:**
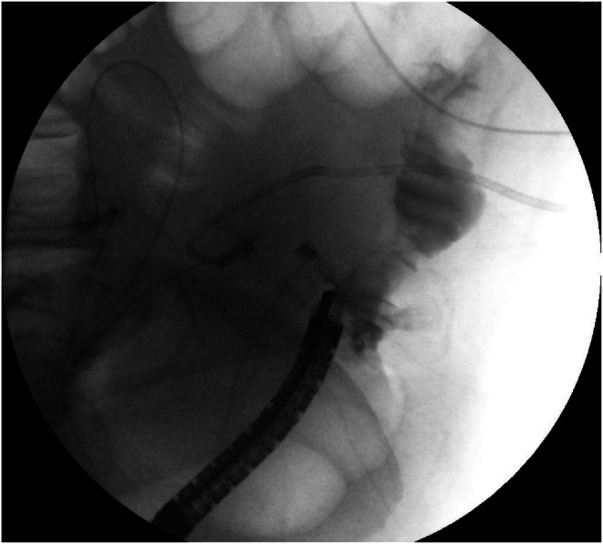
Contrast leaked through the fistula showed a short sinus.

**Figure 2:**
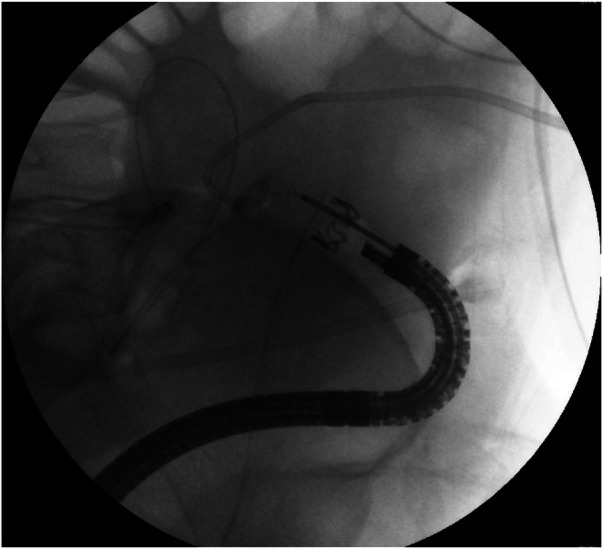
Successful anchoring of the fistula opening hole via OTSC.

**Figure 3:**
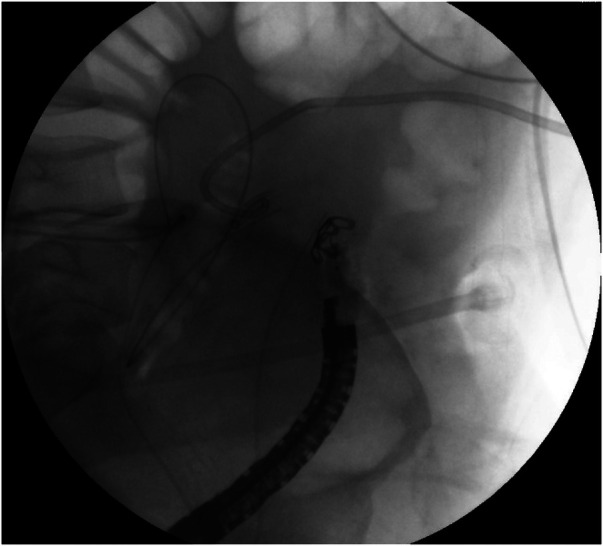
Successful anchoring of the fistula opening hole via OTSC.

After discussion with the patient, an endoscopic approach was selected to avoid surgical re-intervention. On postoperative day 35, endoscopy identified the fistulous opening 33 cm from the anal verge (Supplementary Figures S1 and Figure 1). Closure was achieved using a traumatic-type OTSC 220tt system (Ovesco Endoscopy GmbH, Tübingen, Germany) (Figures 2, 3). No additional defects were visualized following clip deployment.

**Figure 4:**
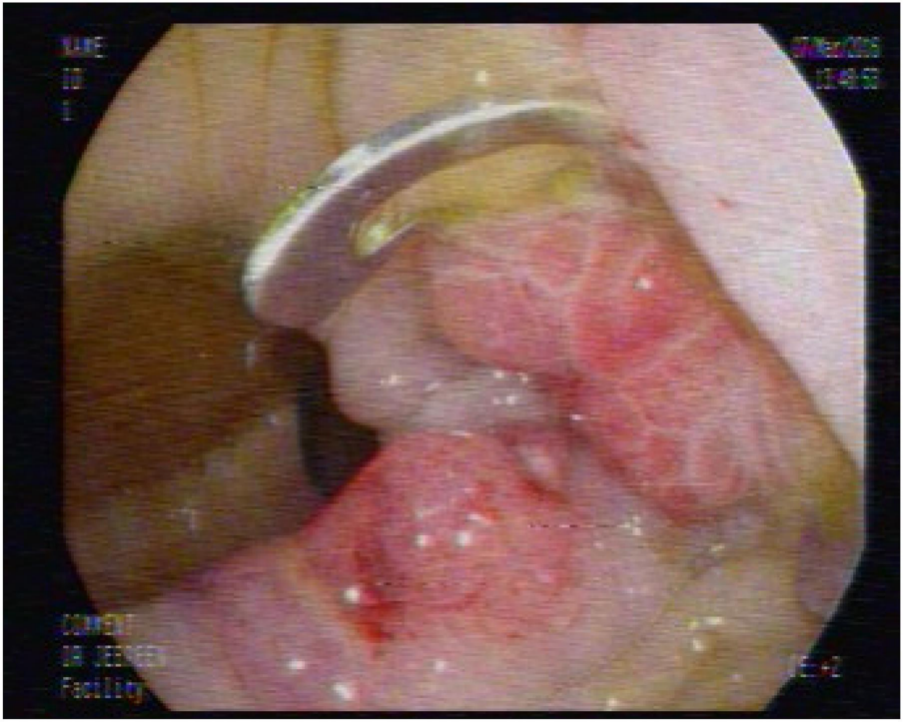
No opening at the site of the previous fistula was found 8 days post-procedure.

**Figure 5:**
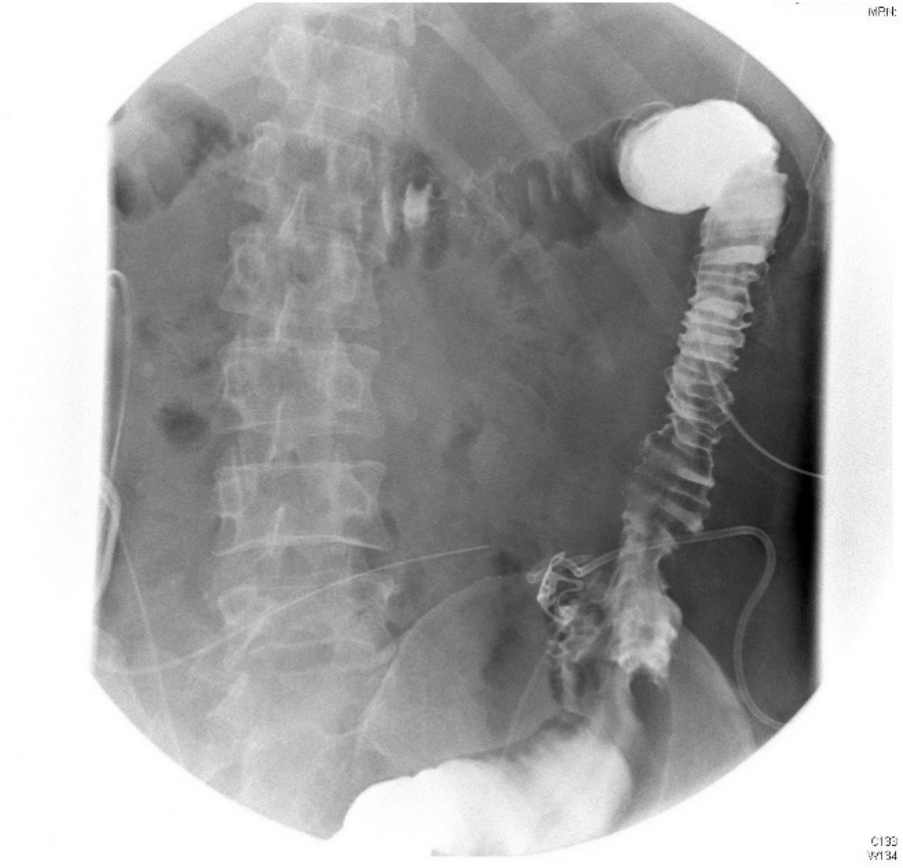
Gastrograffin enema confirmed the absence of any leak.

Eight days later, colonoscopy and gastrografin enema confirmed complete closure of the anastomotic leak (Figures 4, 5). The patient was discharged 10 days after the procedure in stable condition, without complications or recurrence.

## Discussion

Anastomotic leakage remains one of the most feared complications following colorectal surgery due to its association with sepsis, prolonged hospitalization, and impaired functional outcomes. To align with standardized reporting, leaks are described according to the International Study Group of Rectal Cancer (ISREC) definition and graded using the Clavien–Dindo classification, rather than subclinical/clinical terminology.

In patients with controlled leakage and adequate drainage, non-operative supportive management is generally the initial approach, as approximately half of colocutaneous fistulas may close spontaneously within a mean duration of 30 days (7). However, failure of conservative management or persistence of a major defect typically necessitates escalation of therapy (8).

For sigmoid or rectal anastomotic leaks, Hartmann's procedure has historically been considered a safe surgical option (6). However, re-operation is associated with substantial morbidity, particularly in critically ill or polytrauma patients.

Recent advances in therapeutic endoscopy have introduced minimally invasive alternatives. The OTSC system enables full-thickness closure of gastrointestinal wall defects and has demonstrated increasing utility in managing anastomotic dehiscence, leaks, and fistulas (4).

In the present case, I selected OTSC over through-the-scope clips or endoscopic suturing due to its superior compression force, ability to capture fibrotic and inflamed postoperative tissue, and higher likelihood of durable closure. The traumatic-type OTSC with sharp teeth was deliberately chosen to optimize tissue grip in a chronic, indurated fistula tract.

Several studies have reported successful OTSC use in chronic gastrocutaneous, tracheoesophageal, gastric, and coloenteric fistulas (9–11). More recently, successful management of anastomotic dehiscence following right hemicolectomy has been reported (12). A large systematic review demonstrated an overall OTSC success rate of 78%, including 66% for anastomotic dehiscence, with a low severe complication rate of 0.59% (13).

While this case involved a colo-sigmoid anastomosis, the applicability of OTSC may be limited in very low colorectal or coloanal anastomoses, irradiated tissue, or severe ischemia. These anatomical and technical factors should be evaluated on an individual basis.

## Conclusion

This case demonstrates the successful use of the over-the-scope clip (OTSC) system for the management of a persistent colonic anastomotic leak following trauma-related large bowel surgery. After failure of conservative management, endoscopic closure with OTSC achieved complete resolution of the leak without the need for surgical re-intervention or fecal diversion. This minimally invasive approach may represent a valuable therapeutic option in carefully selected, clinically stable patients with localized colonic anastomotic defects. Further studies are warranted to better define patient selection criteria and to compare the efficacy and safety of OTSC with other endoscopic and surgical management strategies.

## Data Availability

The original contributions presented in the study are included in the article/Supplementary Material, further inquiries can be directed to the corresponding author.
