# Supplementary figures and images for: Case Report: Use of the over-the-scope clipping system in a trauma patient for a large bowel anastomotic leak
Source: Front Surg. 2026 May 20;13:1695835. doi: 10.3389/fsurg.2026.1695835 (PMC13230035; doi:10.3389/fsurg.2026.1695835)

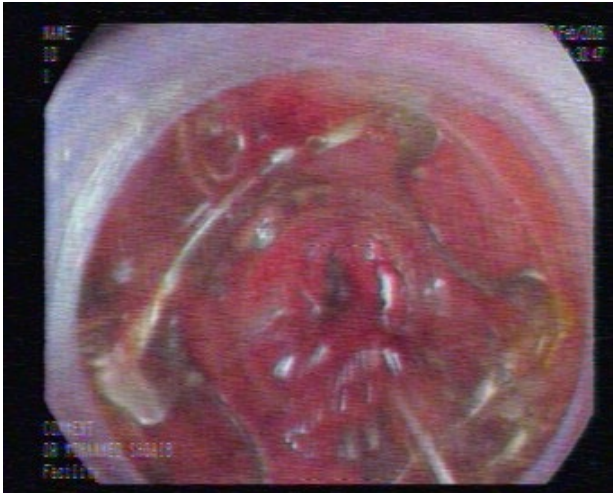

Figure s1. The opening of the fistula noticed at 33 cm from the anal verge.

Supplement: Supplementary file 1 [file Image1.pdf]
